# Ligand and Structure-Based In Silico Determination of the Most Promising SARS-CoV-2 nsp16-nsp10 2′-*o*-Methyltransferase Complex Inhibitors among 3009 FDA Approved Drugs

**DOI:** 10.3390/molecules27072287

**Published:** 2022-03-31

**Authors:** Ibrahim H. Eissa, Mohamed S. Alesawy, Abdulrahman M. Saleh, Eslam B. Elkaeed, Bshra A. Alsfouk, Abdul-Aziz M. M. El-Attar, Ahmed M. Metwaly

**Affiliations:** 1 Pharmaceutical Medicinal Chemistry and Drug Design Department, Faculty of Pharmacy (Boys), Al-Azhar University, Cairo 11884, Egypt; mohammedalesawy@azhar.edu.eg (M.S.A.); abdo.saleh240@azhar.edu.eg (A.M.S.); 2 Department of Pharmaceutical Sciences, College of Pharmacy, Almaarefa University, Riyadh 13713, Saudi Arabia; ikaeed@mcst.edu.sa; 3 Department of Pharmaceutical Sciences, College of Pharmacy, Princess Nourah Bint Abdulrahman University, P.O. Box 84428, Riyadh 11671, Saudi Arabia; baalsfouk@pnu.edu.sa; 4 Pharmaceutical Analytical Chemistry Department, Faculty of Pharmacy, Al-Azhar University, Cairo 11884, Egypt; zizoalattar@yahoo.com; 5 Pharmacognosy and Medicinal Plants Department, Faculty of Pharmacy (Boys), Al-Azhar University, Cairo 11884, Egypt; 6 Biopharmaceutical Products Research Department, Genetic Engineering and Biotechnology Research Institute, City of Scientific Research and Technological Applications (SRTA-City), Alexandria 21934, Egypt

**Keywords:** SARS-CoV-2 nsp16-nsp10 2′-*o*-methyltransferase, FDA approved drugs, molecular fingerprints, structural similarity, molecular docking, MD simulations, MMPBSA

## Abstract

As a continuation of our earlier work against SARS-CoV-2, seven FDA-approved drugs were designated as the best SARS-CoV-2 nsp16-nsp10 2′-*o*-methyltransferase (2′OMTase) inhibitors through 3009 compounds. The in silico inhibitory potential of the examined compounds against SARS-CoV-2 nsp16-nsp10 2′-*o*-methyltransferase (PDB ID: (6W4H) was conducted through a multi-step screening approach. At the beginning, molecular fingerprints experiment with **SAM** (*S*-Adenosylmethionine), the co-crystallized ligand of the targeted enzyme, unveiled the resemblance of 147 drugs. Then, a structural similarity experiment recommended 26 compounds. Therefore, the 26 compounds were docked against 2′OMTase to reveal the potential inhibitory effect of seven promising compounds (Protirelin, (**1187**), Calcium folinate (**1913**), Raltegravir (**1995**), Regadenoson (**2176**), Ertapenem (**2396**), Methylergometrine (**2532**), and Thiamine pyrophosphate hydrochloride (**2612**)). Out of the docked ligands, Ertapenem (**2396**) showed an ideal binding mode like that of the co-crystallized ligand (**SAM**). It occupied all sub-pockets of the active site and bound the crucial amino acids. Accordingly, some MD simulation experiments (RMSD, RMSF, R_g_, SASA, and H-bonding) have been conducted for the 2′OMTase—Ertapenem complex over 100 ns. The performed MD experiments verified the correct binding mode of Ertapenem against 2′OMTase exhibiting low energy and optimal dynamics. Finally, MM-PBSA studies indicated that Ertapenem bonded advantageously to the targeted protein with a free energy value of −43 KJ/mol. Furthermore, the binding free energy analysis revealed the essential amino acids of 2′OMTase that served positively to the binding. The achieved results bring hope to find a treatment for COVID-19 via in vitro and in vivo studies for the pointed compounds.

## 1. Introduction

The WHO, addressed on 16 February 2022, confirmed that the worldwide infections of COVID-19 were 414,525,183. Grievously, this number includes 5,832,333 deaths [1]. Although 10,227,670,521 vaccinations have been administered [1], the virus can still infect and spread widely [2]. Responding to these numbers, massive work is demanded from scientists all over the world to find a cure.

The regular process of new drug discovery is highly expensive and takes much time. The average required time for the complete development of a new drug is about 12 years, with a cost of 2.6 billion USD [3]. In contrast, drug repurposing or repositioning is a much faster technique in which the exploration of new pharmacological use for an old or existing drug occurs [4]. The strategy of drug repurposing was applied successfully in the discovery of anti-cancer [5], COVID-19 [6], anti-inflammatory [7], antibacterial [8], anti-parasitic [9], and anti-viral [10] drugs. 

The tremendous applications of computational chemistry in drug discovery are due to different factors. First, the exploration of accurate 3D structures of different protein targets in the human body [11]. Second, the vast advancements in the fields of computer hardware and software [12]. Finally, the development of structure–activity relationship (SAR) principles [13]. Consequently, computational chemistry methods were applied to estimate various pharmacodynamic and pharmacokinetic parameters that relate the chemical structure of compounds to its activity and also to characterize the interaction of compounds with biological targets such as structure similarity [14], molecular fingerprints [15], QSAR [16], pharmacophores [17], homology models [18], molecular modeling [19], drug molecular design [20], rational drug design [21,22], molecular docking [23], MD simulations [24], absorption [25], distribution [26], metabolism [27], excretion [28], and toxicity properties [29], as well as physicochemical characterization [30] and DFT [31].

In this regard, our team employed the strategies of computer-based chemistry to discover the potential inhibitive effects of the secondary metabolites of *Asteriscus hierochunticus* [32], *Monanchora* sp. [33], *Artemisia sublessingiana* [34], and *Artemisia* sp. [35], as well as 69 isoflavonoids [36] against SARS-CoV-2. Additionally, we designed a multi-step in silico selection method to prime the most active inhibitor drugs against a SASRS-CoV-2 protein amongst a vast number of compounds. As an exemplification, amongst 310 natural metabolite and 69 semisynthetic compounds, the highest potential inhibitors against SARS-CoV-2 nsp10 [37] and the SARS-CoV-2 PLpro [38], respectively, were decided

In this research, a panel of 3009 FDA-approved compounds was retrieved from the internet [39] to be screened depending on various computational methods to distinguish the most potent SARS-CoV-2 nsp16-nsp10 2′-*o*-methyltransferase complex inhibitor.

The starting point in our study was (S-Adenosylmethionine, **SAM**), the co-crystallized ligand of the essential COVID-19 protein, 2′OMTase (PDB ID: (6W4H), that showed high binding affinity against it. Firstly, the selected compounds were subjected to two ligand-based computational techniques (molecular fingerprints and similarity) successively to select the most similar candidates to **SAM**. Then, several structure-based computational methods (molecular docking and MD simulations) were conducted to confirm the binding modes, energies, and dynamic behaviors of the singled-out candidates. 

## 2. Results and Discussion

### 2.1. Filter Using Fingerprint

Molecular fingerprint is a ligand-based computational (*in silico*) computational technique. This approach can predict the biological activity of a molecule based on its chemical structure [40]. The scientific base of ligand-based calculations is influenced by the principles of target– structure–activity relationships (SAR). It can set a relationship between the measured bio response/s exerted by a molecule and its chemical structure. Accordingly, compounds with similar chemical structures are expected to exert similar activities [41]. 

A co-crystallized ligand is one that exerts an excellent binding affinity with the corresponding protein forming a crystallizable ligand–protein complex [42]. In accordance, the chemical structure of that ligand could be employed as a model to design and develop an inhibitor that can bind strongly to the target protein. The molecular fingerprints study was performed using Discovery Studio against **SAM**. The experiment examined the next variables: H-bond acceptor and donor [43], charge [44], hybridization [45], positive and negative ionizable [46], halogen, aromatic, or none of the above besides the ALogP of atoms and fragments.

In structural terms, the chemical structures of the examined molecules are encoded and transformed binary bit strings (sequences of 0′s and 1′s). Every bit corresponds to a “pre-defined/determined” structural descriptor or feature of substructure or fragment. If the examined molecule has that feature, the bit position that corresponds to this descriptor is set to 1 (ON). If it is absent, it is set to 0 (OFF) [47]. 

SA describes the number bits that were computed in the FDA-approved drugs and the **SAM**. SB identifies the number bits that were found in the FDA-approved drugs, but not **SAM**. SC refers to the number bits that were discovered in **SAM**, but not in the FDA-approved drugs.

The study (Table 1) favored 147 compounds. These compounds showed the highest fingerprint similarity with **SAM**.

### 2.2. Molecular Similarity

The connection between chemical structures and biological activities of different compounds has always been an interesting area for research [48]. Consequently, the implementation of different molecular similarity strategies in drug design and development have been competently increased effectively [49]. Many descriptors have been considered in molecular similarity studies. 

The examined descriptors are of a molecular type, such as molecular weight (M.W.) [50], hydrogen bond donors (HBA) [51], hydrogen bond acceptors (HBD) [52], partition coefficient (ALog p), which is the ratio of the concentration of a substance in the lipid phase to the concentration in the aqueous phase when the two concentrations are at equilibrium [53], number of rotatable bonds [54], number of rings, and aromatic rings [55], as well as the molecular fractional polar surface area (MFPSA) [56]. The examined compound is represented as a binary array (number of binary bits) to be computed. 

The mentioned descriptors were calculated for the FDA-approved drugs then compared with the co-crystallized ligand of 2′OMTase (**SAM**) using Discovery studio software. 

**Figure 1:**
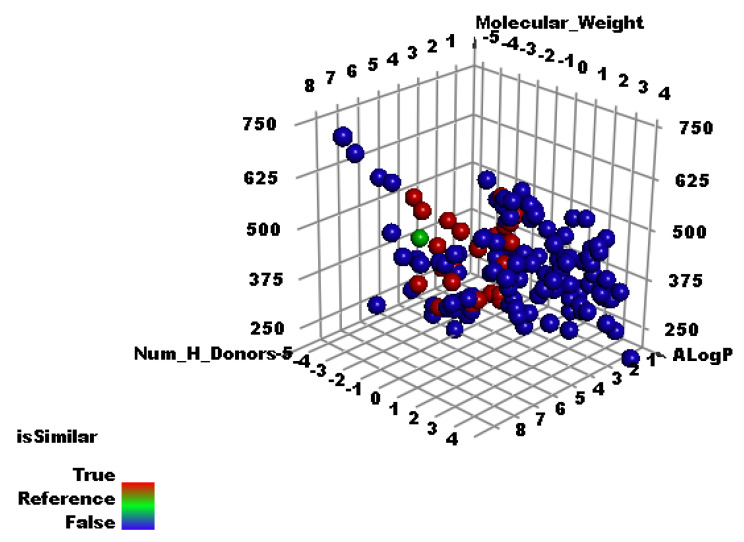
The molecular similarity of the examined compounds and **SAM**.

**Figure 2:**
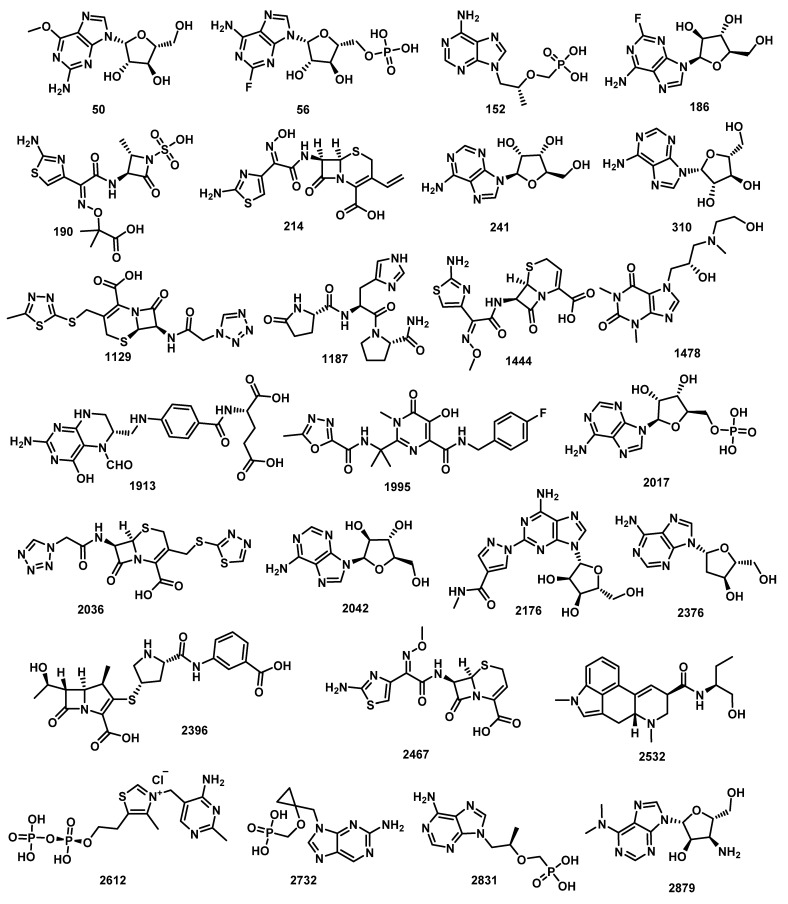
Twenty-six compounds with good molecular similarity with the co-crystallized ligand (**SAM**) of 2′OMTase (PDB ID: (6W4H).

Figure 1 represented the co-crystalized ligand (**SAM**) (red ball), compounds with good similarities (green balls), and compounds with diminished similarities (blue balls). The degree of molecular likeness or similarity between two compounds depends on a similarity coefficient that is utilized to compute a quantitative score. That calculated score is equivalent to the degree of similarity and is based on the computed values of several structural descriptors. Similarity between two compounds is inversely proportional to the calculated distance between them in the descriptor space [57]. In this work, the distances between the several descriptors were computed to determine descriptor similarity among test compounds and **SAM** [58]. The computed distances describe the shortest distance between two points. Typed graph distances (Figure 1) show the overall similarity of behavior of the FDA-approved drugs compared to **SAM**. The study preferred 26 compounds among the most suitable 30 metabolites (Figure 1 and Figure 2, and Table 2).

### 2.3. Docking Studies

Docking studies of the tested compounds were conducted using the MOE (Molecular Operating Environment) software [58] to understand the proposed binding mode and the orientations of such compounds with the prospective target 2′OMTase (PDB ID: (6W4H)).

**Figure 3:**
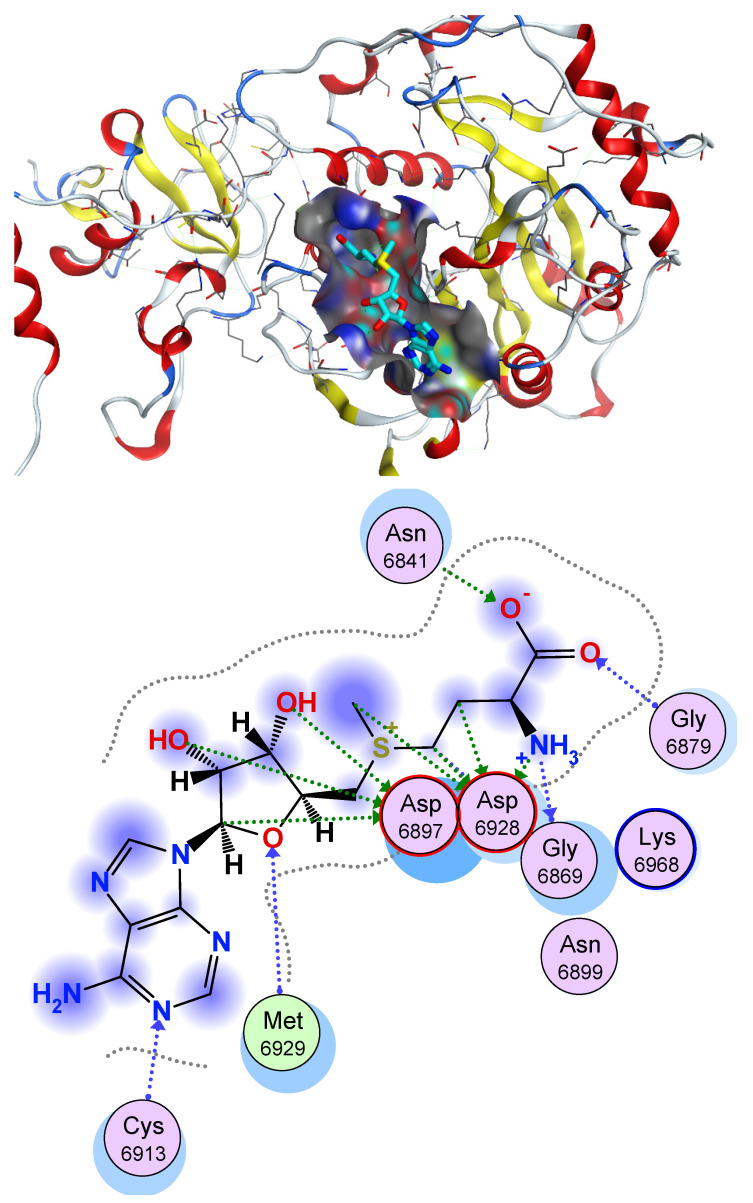
Active site (3D and 2D) of 2′OMTase (PDB ID: (6W4H)).

The active site of 2′OMTase consists of some crucial amino acids which can form hydrogen bonds with the active ligands. These amino acids include: Asn6841, Gly6879, Gly6869, Asp6928, Asp6897, Met6929, and Cys6913. In addition, there are some hydrophobic amino acids which can be incorporated in hydrophobic attractions with the active ligand and the hydrophobic amnio acids such as Leu6898 and Met6929 (Figure 3).

**Figure 4:**
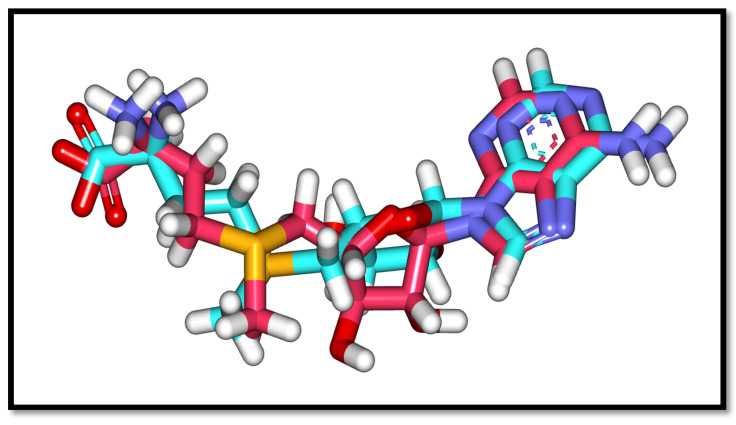
Alignment of the co-crystallized ligand (turquoise) and the docking pose (rose) of the same ligand (**SAM**) in the active site of 2′OMTase.

The co-crystalized ligand s-adenosylmethionine (**SAM**) was used as a reference compound. First, the validation process was carried out to confirm the validity of the docking algorithm in obtaining accurate docking results. This was achieved by redocking the co-crystallized ligand (**SAM**) with 2′OMTase. The obtained low values of root mean square deviation (RMSD = 1.15 Å) between the native and redocked pose, in addition to the symmetrical superimposition in orientation between both the native (turquoise) and redocked (magenta) co-crystallized poses in Figure 4, guaranteed the valid performance of the docking protocol [36,38], in addition to the docking algorithm’s capability to obtain the reported binding mode of the co-crystalized ligand S-adenosylmethionine (**SAM**) [59]. 

In comparing the tested compounds, the binding free energy (ΔG) between the docked molecules and the active site, and also the proper binding mode, were properly considered. The estimated (ΔG) (binding free energies) of the investigated drugs and the reference molecule (**SAM**) against the 2′OMTase are presented in Table 3.

**Figure 5:**
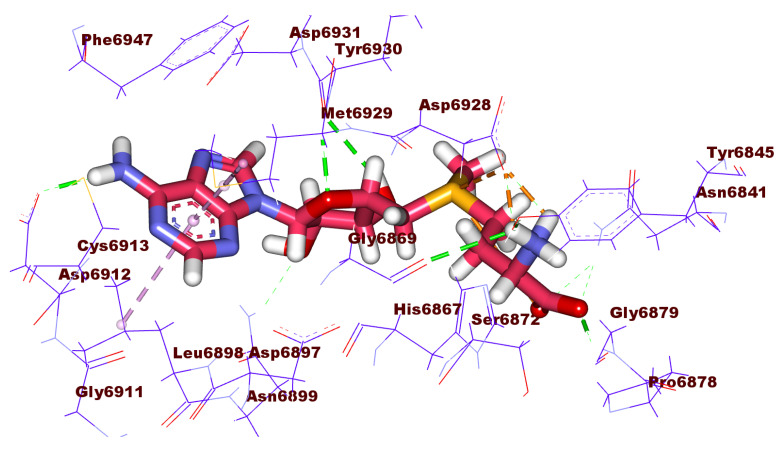

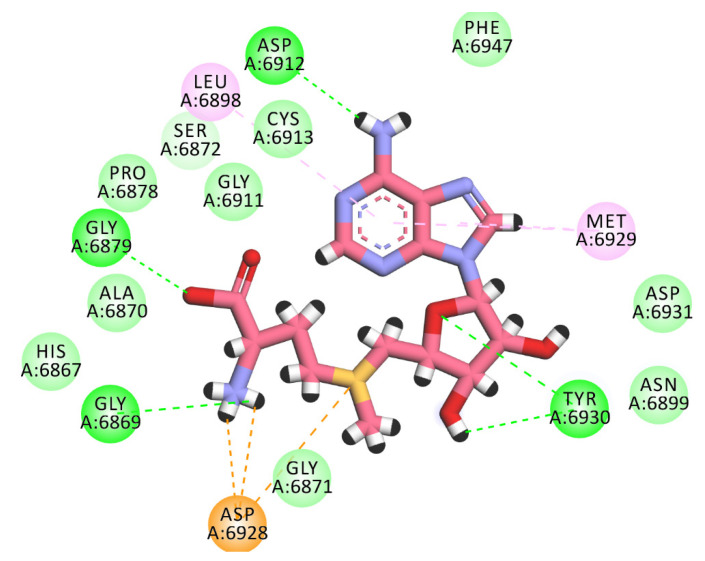
3D and 2D binding mode of the redocked ligand (**SAM**) in the active site of the target protein.

The predicted binding mode of the redocked ligand (**SAM**) yielded an affinity value of −21.52 kcal/mol. It interacted with its 6-amino-purin moiety and formed one hydrogen bond with Asp6912, in addition to hydrophobic interactions with Leu6898 and Met6929. Moreover, the di hydroxy tetrahydrofuran moiety formed two hydrogen bonds with Tyr6930, and the sulfur atom was involved in electrostatic interaction with Asp6928. Additionally, the terminal NH_2_ group was found to form one hydrogen bond with Gly6869, and two electrostatic interactions with Asp6928. Finally, the terminal carboxylic group formed one hydrogen bond with Gly6879 (Figure 5). 

From the tested compounds, seven members showed good binding mode with high binding energy. These compounds are **1187** (Protirelin), **1913** (Calcium folinate), **1995** (Raltegravir), **2176** (Regadenoson), **2396** (Ertapenem), **2532** (Methylergometrine), and **2612** (Methylergometrine).

**Figure 6:**
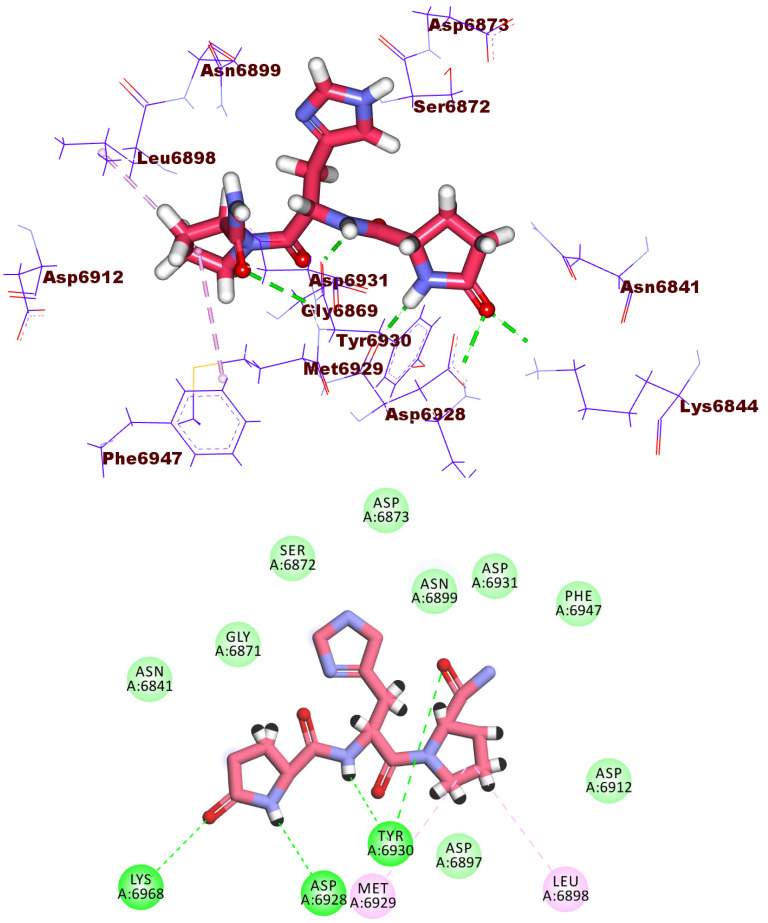
3D and 2D binding mode of compound **1187** in the active site of the target protein.

Compound **1187** has a docking score of −18.68 kcal/mol and formed four hydrogen bonds with the crucial amino acids in the active site of the 2′OMTase enzyme. The pyrrolidin-2-one moiety formed two hydrogen bonds with Asp6928 and Lys6968 via its NH and C=O groups, respectively. Furthermore, the NH group of the central amide moiety formed one hydrogen bond with Tyr6930. Moreover, the (S)-pyrrolidine-2-carboxamide moiety formed one hydrogen with Tyr6930 and two hydrophobic bonds with Met6929 and Leu6898 (Figure 6).

**Figure 7:**
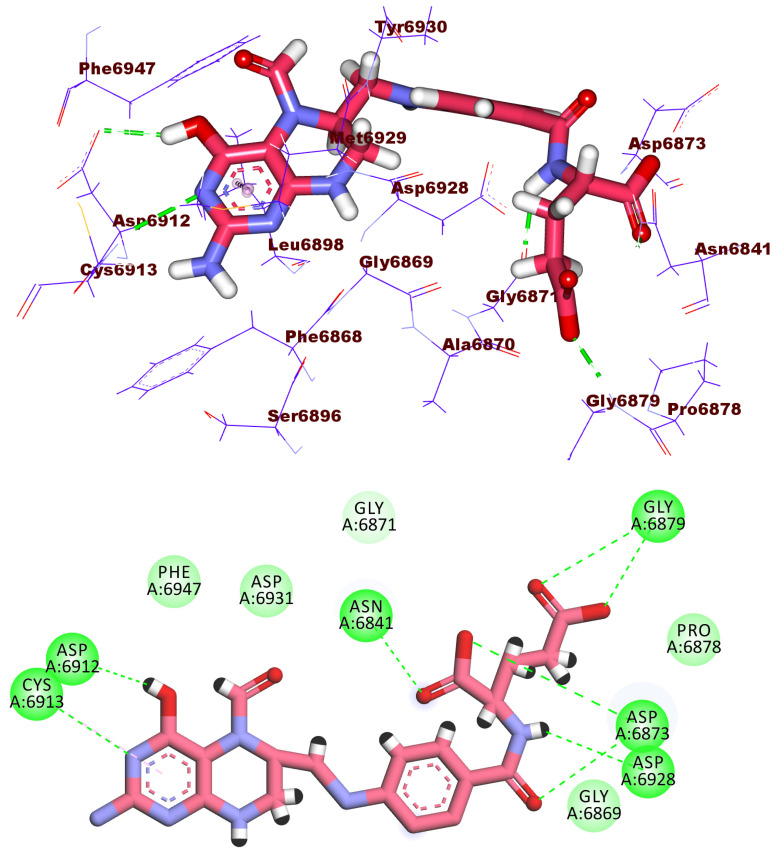
3D and 2D binding mode of compound **1913** in the active site of the target protein.

Compound **1913** has a docking score of −19.09 kcal/mol, forming six hydrogen bonds within the active site. The 2-amino-4-hydroxy-7,8-dihydropteridine-5(6*H*)-carbaldehyde moiety formed three hydrogen bonds with Cys6913, Gly6911, and Asp6912 via its NH_2_, OH groups, and the hetero nitrogen atom at 3-position. In addition, the glutamic acid moiety formed three hydrogen bonds with Asn6841, Gly6879, and Gly6871. Moreover, a carboxylate group of glutamic acid moiety formed one electrostatic interaction with Asp6873 (Figure 7).

**Figure 8:**
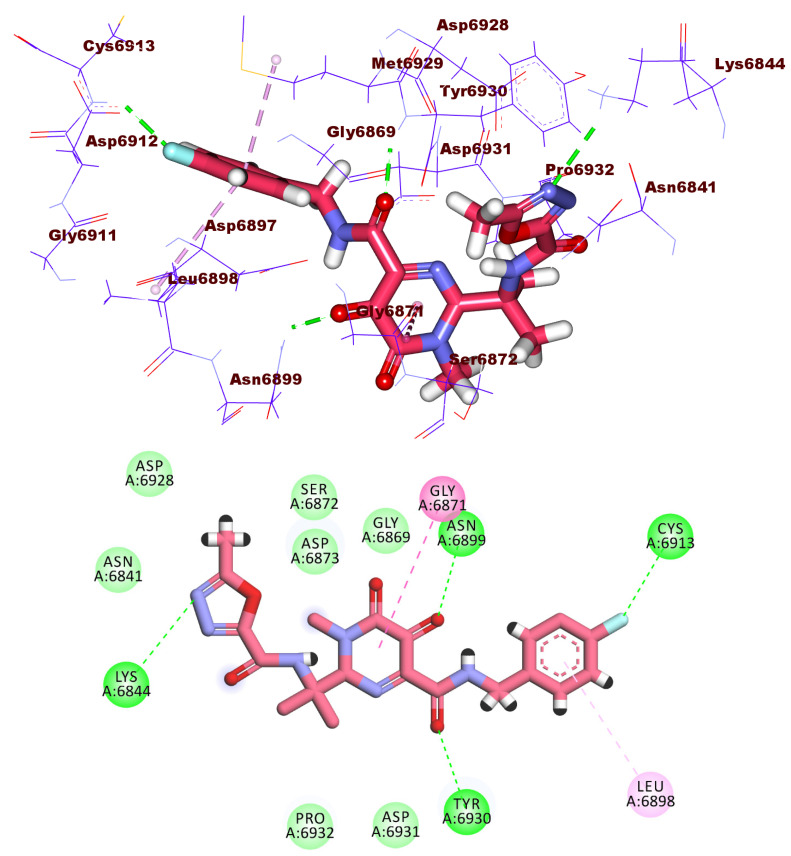
3D and 2D binding mode of compound **1995** in the active site of the target protein.

With a docking score of −21.07 kcal/mol, compound **1995** fit well into the active site of the 2′OMTase enzyme and formed four hydrogen bonds. The fluorobenzene formed one hydrogen bond with Cys6913 and one hydrophobic interaction with Leu6898. The carbonyl group of the amide moiety formed one hydrogen bond with Tyr6930. The carbonyl group of 5-hydroxy-3-methylpyrimidin-4(3*H*)-one moiety formed one hydrogen bond with Asn6899. In addition, the 5-hydroxy-3-methylpyrimidin-4(3*H*)-one moiety formed hydrophobic bond with Gly6871. The NH group of 2-methyl-1,3,4-oxadiazole moiety formed one hydrogen bond with Lys6844 (Figure 8). 

**Figure 9:**
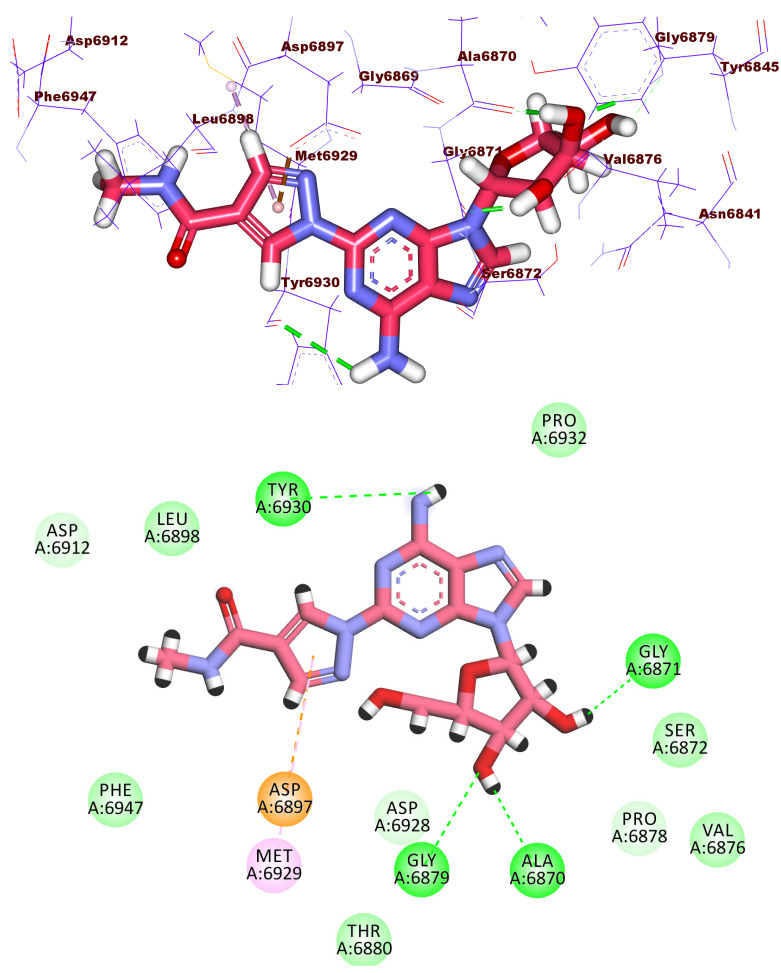
3D and 2D binding mode of compound **2176** in the active site of the target protein.

Compound **2176** showed a binding energy of −18.54 kcal/mol. This compound formed four hydrogen bonds in the active site of the target protein. The ribose sugar moiety formed three hydrogen bonds with Gly6879, Ala6870, and Gly6871. Furthermore, the NH group of the 9H-purin-6-amine moiety formed a hydrogen bond with Ty6930. Moreover, the N-methyl-1H-pyrazole-4-carboxamide moiety was incorporated in hydrophobic interaction with Met6929 and one electrostatic interaction with Asp6897 (Figure 9).

**Figure 10:**
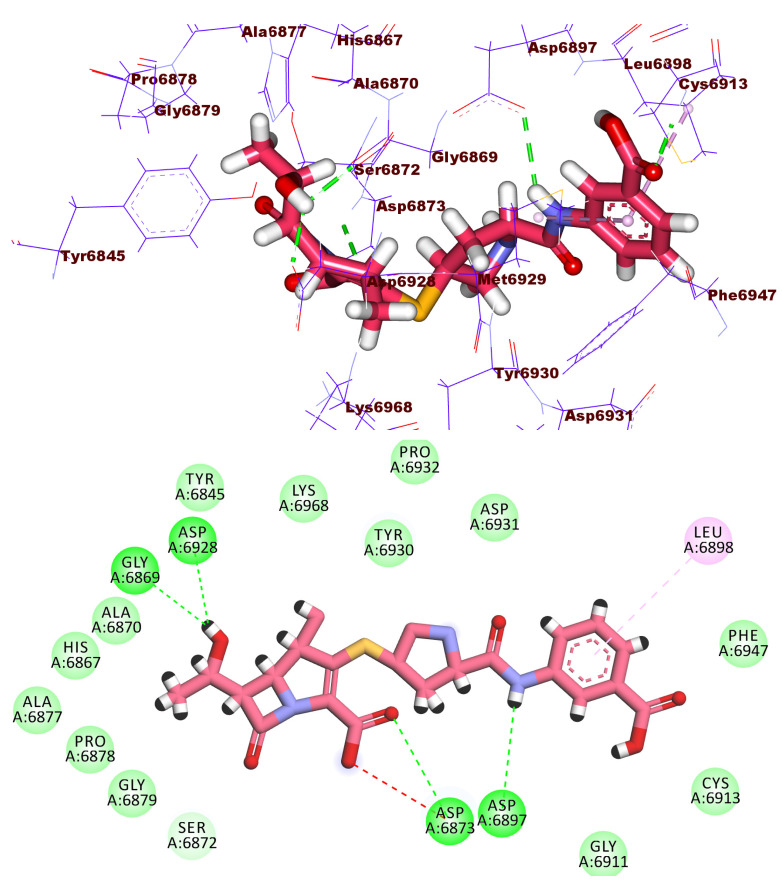
3D and 2D binding mode of compound **2396** in the active site of the target protein.

Compound **2396** (Ertapenem) has a docking score of −20.73 kcal/mol and created five hydrogen bonds with the crucial amino acids in the active site of the 2′OMTase enzyme. The benzoic acid moiety formed one hydrogen bond with Cys6913 via its carboxylic group, and two hydrophobic interactions with Met6929 and Leu6898. Furthermore, the NH group formed another hydrogen bond with Asp6897. Moreover, the carboxylate group at 2-position of 1-azabicyclo[3.2.0]hept-2-ene moiety formed one hydrogen and one electrostatic bond with Asp6873. The terminal hydroxyl group formed two hydrogen bonds with Asp6928 and Gly6869 (Figure 10). Although Ertapenem showed a binding energy less than Raltegravir, it showed an ideal binding mode like that of the co-crystallized ligand (**SAM**). It occupied all sub pockets of the active site and bound the crucial amino acids. Accordingly, it was used for further in silico testing via MD simulations.

**Figure 11:**
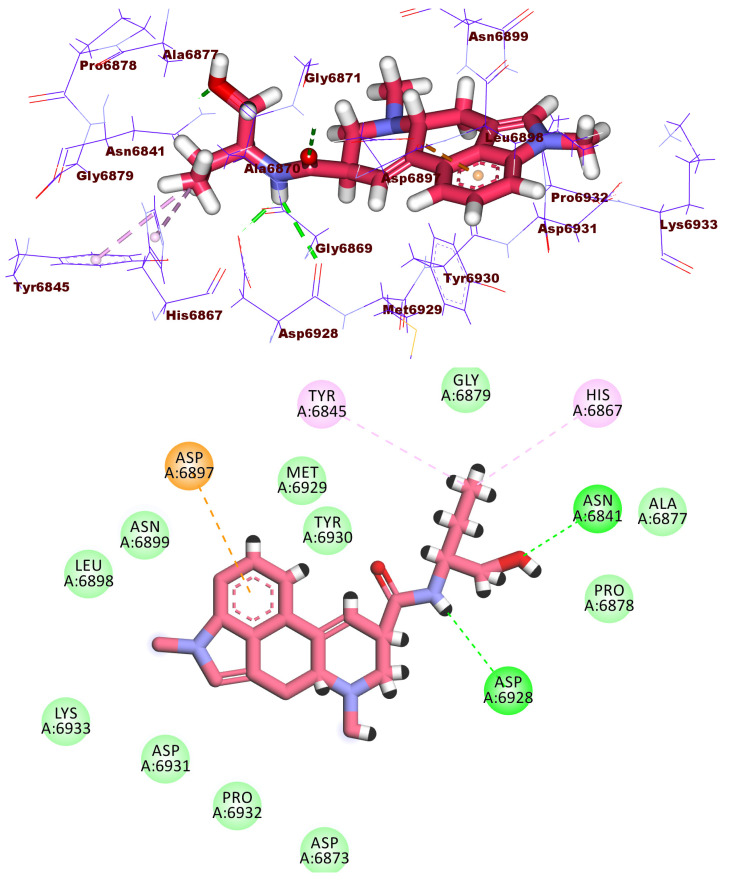
3D and 2D binding mode of compound **2532** in the active site of the target protein.

The binding mode of compound **2532** (affinity value of −20.46 kcal/mol), which is extremely close to ligand **SAM**, revealed that the amide group formed two hydrogen bonds with fundamental amino acids Asp6928 and Gly6871. In addition, the OH group formed another hydrogen bond with the amino acid Asn6841. Furthermore, the terminal ethyl moiety was incorporated in hydrophobic interaction with His6867 and Tyr6845. The phenyl ring formed an electrostatic attraction with Asp6897 (Figure 11).

**Figure 12:**
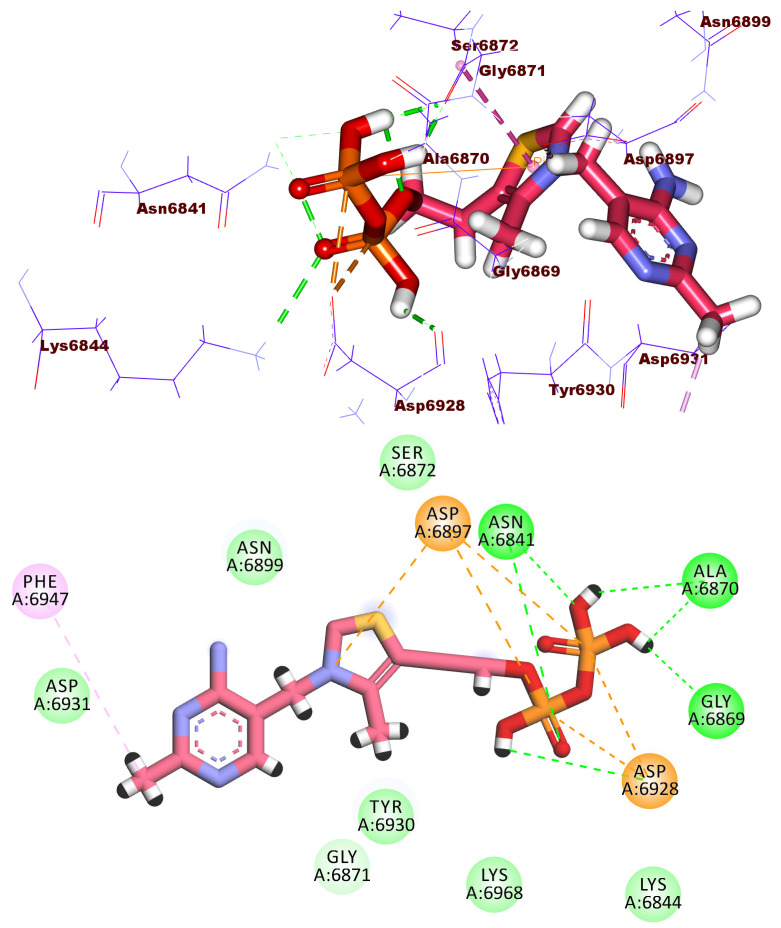
3D and 2D binding mode of compound **2612** in the active site of the target protein.

As demonstrated in Figure 12, compound **2612** had a high potential binding affinity (ΔG = −18.03 kcal/mol) with 2′OMTase enzyme active site. The strong binding affinity is assumed to be due to the formation of four hydrogen bonds in addition to many hydrophobic and electrostatic attractions. The terminal diphosphate moiety formed four hydrogen bonds with Gly6871, and Asn6841. It also formed three electrostatic attractions with Asp6928. In addition, the 4-methylthiazol-3-ium moiety formed a hydrophobic interaction with Gly6871 and an electrostatic attraction with Asp6897. Furthermore, the 2-methylpyrimidin-4-amine moiety was incorporated in a hydrophobic attraction with Phe6947.

### 2.4. Molecular Dynamic Simulation

Molecular dynamics (MD) simulations studies can be applied to examine almost every kind of biomacromolecule (protein, nucleic acid, or carbohydrate) of biological significance [60]. The MD experiments can afford abundant information regarding the dynamic structural of the studied system [61]. Additionally, it contributes large amounts of energetic data. Such data are essential to understand the structure–function relationship of the examined ligand, its target protein, as well as the protein–ligand interactions. Correspondingly, MD studies could be a vital guide in the drug design and discovery processes [62].

**Figure 13:**
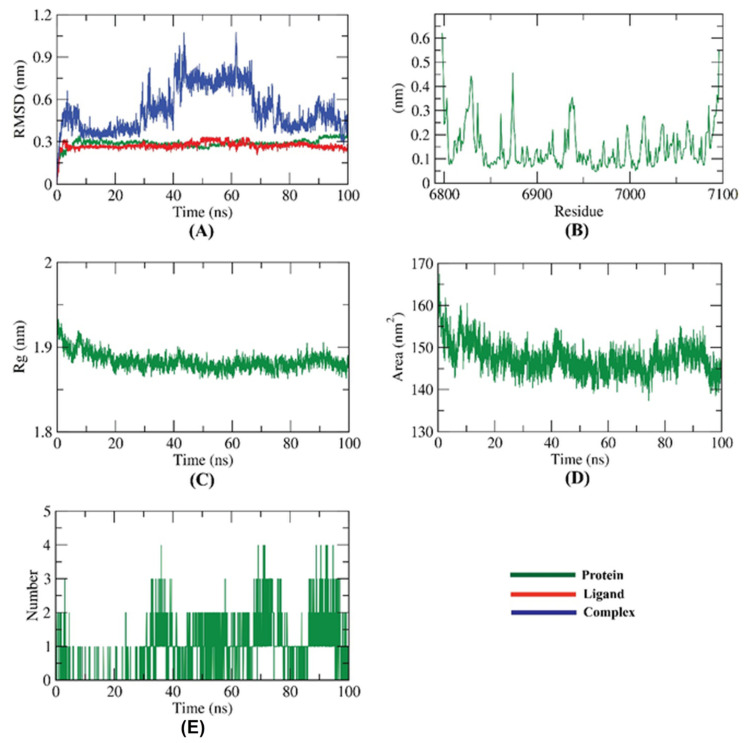
Results of M D simulations of 2′OMTase—Ertapenem complex; (**A**) RMSD, (**B**) RMSF, (**C**) R_g_, (**D**) SASA, and (**E**) H-bonding.

The dynamic, as well as conformational shifts of backbone atoms of 2′OMTase, Ertapenem in addition to 2′OMTase—Ertapenem complex, were estimated through the calculation of the root mean square deviation (RMSD) to distinguish the stability of the examined molecules before and after binding. RMSD investigation demonstrates both of conformational and dynamics changes [63] that occur after binding. Excitingly, the 2′OMTase—Ertapenem complex demonstrated low RMSD values with slight fluctuations from 40–70 ns~ and was stabilized later until the end of the study (Figure 13A) Fortunately, this slight fluctuation did not affect the integrity of the 2′OMTase—Ertapenem complex as the next experiments (R_g_ and SASA) did not record major changes in this time. However, the H-binding showed that the number of H-bonds decreased in this period (40–70 ns), from 3–4 bonds to 2 bonds. The study demonstrated that the number of H-bonds became 3–4 again after 70 ns. The flexibility of the evaluated complex was measured in the terms of RMSF to identify the fluctuated region of 2′OMTase during the 100 ns of the simulation. Favorably, the binding of Ertapenem does not make 2′OMTase very flexible (Figure 13B). The compactness of the 2′OMTase—Ertapenem complex was investigated by the computation of radius of gyration (R_g_) of the evaluated enzyme. Complementarily, the Rg exhibited was noticed to be of lower value during the 100 ns of the experiment compared to the starting time (Figure 13C). In a similar manner, SASA (solvent accessible surface area) denotes the interaction between 2′OMTase—Ertapenem complex, and the surrounding solvents was measured. SASA value is an excellent indicator to the conformational changes that occurred during the simulation experiment because of binding interactions. Of note, the surface area of 2′OMTase (Figure 13D) displayed a considerable reduction in SASA values through the simulation time compared to the starting point. Finally, hydrogen bonding, as an essential factor in the binding of 2′OMTase—Ertapenem complex, was estimated. The greatest number of H-bonds that formed between 2′OMTase—Ertapenem complex was up to three H-bonds (Figure 13E).

### 2.5. Molecular Mechanics Poisson-Boltzmann Surface Area (MM-PBSA) Studies

**Figure 14:**
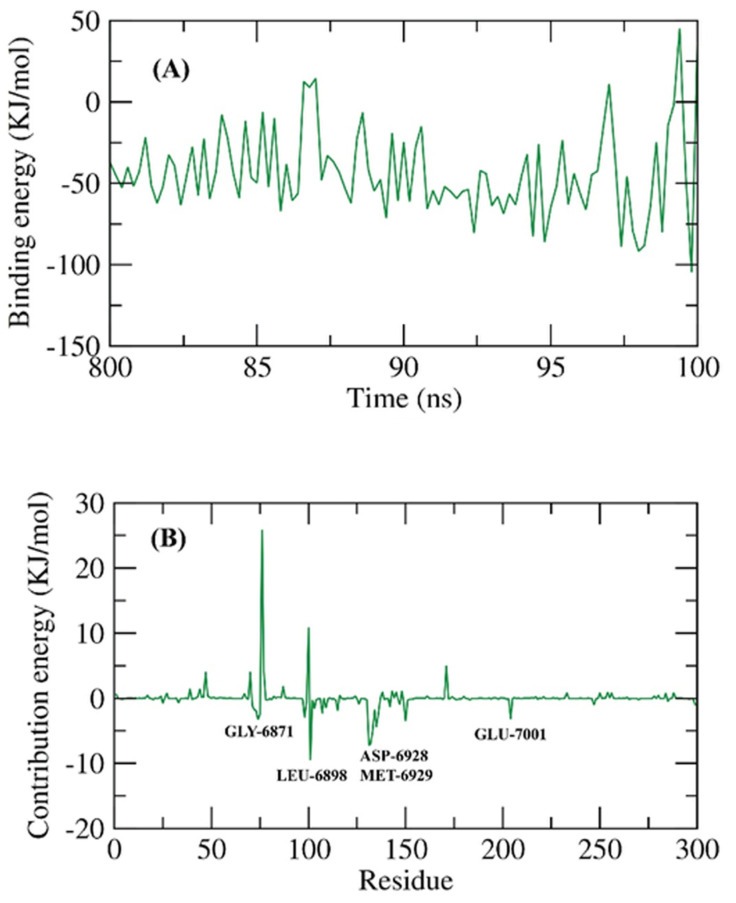
MM-PBSA study of 2′OMTase—Ertapenem; (**A**): total binding free energy, (**B**): analyzed binding free energy per amino acid residue.

The binding free energy of 2′OMTase—Ertapenem complex was investigated in the last 20 ns of the MD run with an interval of 100 ps from the produced MD trajectories. The MM/PBSA method was utilized with the MmPbSaStat.py script to compute the average free binding energy as well as its standard deviation/error. Interestingly, as shown in Figure 14A, Ertapenem demonstrated a low binding free energy with a value of −43 KJ/mol (equivalent to −10.28 kcal/mol) with 2′OMTase. The share of the different amino acid residues of 2′OMTase in respect of the binding energy compared to the binding with Ertapenem. Total binding free energy decomposing of the 2′OMTase—Ertapenem complex into per residue share energy was achieved. The following amino acid residues of 2′OMTase, GLY-6871, LEU-6898, ASP-6928, MET-6829, and GLU-7001, contributed the binding energy with values that are more than −3 KJ/mol (Figure 14B).

## 3. Method

### 3.1. Molecular Similarity Detection

Discovery studio 4.0 software was used (see method part in Supplementary Materials).

### 3.2. Fingerprint Studies

Discovery studio 4.0 software was used (see method part in Supplementary Materials).

### 3.3. Docking Studies

Docking studies were performed against target enzymes using Discovery studio software [64] (see method part in Supplementary Materials).

### 3.4. Molecular Dynamics Simulation

The system was prepared using the web-based CHARMM-GUI [65,66,67] interface utilizing CHARMM36 force field [68] and NAMD 2.13 [69] package. The TIP3P explicit solvation model was used (see Supplementary Materials).

### 3.5. MM-PBSA Studies

The g_mmpbsa package of GROMACS was utilized to calculate the MM/PBSA (See Supplementary Materials).

## 4. Conclusions

Seven FDA-approved compounds (Protirelin, (**1187**), Calcium folinate (**1913**), Raltegravir (**1995**), Regadenoson (**2176**), Ertapenem (**2396**), Methylergometrine (**2532**), and Thiamine pyrophosphate hydrochloride (**2612**), out of 3009 were elected as the strongest 2′OMTase inhibitors. The selection of compounds was based on a multistep in silico study. The utilized studies included molecular fingerprints and structure similarity studies against **SAM**, the co-crystallized ligand of the targeted enzyme in addition to molecular docking studies. Ertapenem (**2396**) was subjected to MD simulation studies (RMSD, RMSF, R_g_, SASA, and H-bonding) for 100 ns, confirming the excellent binding. These encouraging results could be a step to discover an effective cure against COVID-19 through further in vitro and in vivo studies for the pointed candidates.

## Figures and Tables

**Table 1:** Fingerprint similarity between the tested compounds and **SAM**.

Comp.	Similarity	SA	SB	SC	Comp.	Similarity	SA	SB	SC
**SAM**	1	237	0	0	**1670**	0.57	257	214	−20
**4**	0.497396	191	147	46	**1694**	0.5	191	145	46
**42**	0.597	138	−6	99	**1737**	0.506944	146	51	91
**50**	0.651	157	4	80	**1740**	0.491582	146	60	91
**51**	0.581	137	−1	100	**1756**	0.506912	220	197	17
**56**	0.665	171	20	66	**1761**	0.523404	246	233	−9
**58**	0.491525	174	117	63	**1766**	0.54321	176	87	61
**74**	0.495652	171	108	66	**1778**	0.511299	181	117	56
**91**	0.496241	132	29	105	**1792**	0.50211	238	237	−1
**113**	0.485714	170	113	67	**1793**	0.494792	285	339	−48
**130**	0.490463	180	130	57	**1802**	0.56	237	186	0
**152**	0.624	143	−8	94	**1805**	0.501433	175	112	62
**158**	0.5	189	141	48	**1818**	0.508475	210	176	27
**186**	0.644	150	−4	87	**1860**	0.494024	124	14	113
**189**	0.5	122	7	115	**1886**	0.493478	227	223	10
**190**	0.492958	175	118	62	**1911**	0.490683	158	85	79
**214**	0.515723	164	81	73	**1913**	0.494033	207	182	30
**241**	0.717	160	−14	77	**1917**	0.929	235	16	2
**251**	0.490956	190	150	47	**1919**	0.488701	173	117	64
**272**	0.508403	121	1	116	**1927**	0.489796	216	204	21
**281**	0.510806	260	272	−23	**1928**	0.488636	215	203	22
**304**	0.488938	221	215	16	**1932**	0.50303	166	93	71
**310**	0.717	160	−14	77	**1949**	0.505464	185	129	52
**322**	0.486154	158	88	79	**1960**	0.48995	195	161	42
**380**	0.514563	159	72	78	**1993**	0.522599	185	117	52
**390**	0.52862	157	60	80	**1995**	0.488998	200	172	37
**404**	0.535211	190	118	47	**2002**	0.49	147	63	90
**428**	0.498623	181	126	56	**2009**	0.511364	135	27	102
**446**	0.50641	158	75	79	**2017**	0.663	193	54	44
**458**	0.488136	144	58	93	**2023**	0.627	168	31	69
**461**	0.507837	162	82	75	**2024**	0.527378	183	110	54
**470**	0.491803	180	129	57	**2031**	0.57	147	21	90
**515**	0.501493	168	98	69	**2036**	0.487179	171	114	66
**516**	0.561	165	57	72	**2042**	0.664	172	22	65
**539**	0.519149	122	−2	115	**2174**	0.488318	209	191	28
**562**	0.489496	233	239	4	**2176**	0.661	199	64	38
**573**	0.491049	192	154	45	**2232**	0.642	265	176	−28
**598**	0.510504	243	239	−6	**2233**	0.701	202	51	35
**659**	0.540816	159	57	78	**2256**	0.543662	193	118	44
**663**	0.492537	198	165	39	**2268**	0.538776	132	8	105
**672**	0.48913	135	39	102	**2303**	0.503597	210	180	27
**679**	0.501661	151	64	86	**2306**	0.494737	188	143	49
**683**	0.488798	240	254	−3	**2333**	0.494595	183	133	54
**711**	0.566	137	5	100	**2376**	0.643	160	12	77
**723**	0.561	142	16	95	**2396**	0.491525	232	235	5
**736**	0.5	169	101	68	**2410**	0.513587	189	131	48
**753**	0.504425	228	215	9	**2437**	0.489189	181	133	56
**771**	0.486076	192	158	45	**2467**	0.503086	163	87	74
**772**	0.489703	214	200	23	**2483**	0.539185	172	82	65
**781**	0.487603	177	126	60	**2488**	0.542274	186	106	51
**816**	0.497297	184	133	53	**2496**	0.522099	189	125	48
**821**	0.493369	186	140	51	**2501**	0.496711	151	67	86
**824**	0.492958	175	118	62	**2530**	0.495468	164	94	73
**874**	0.553531	243	202	−6	**2532**	0.491667	236	243	1
**919**	0.504032	125	11	112	**2538**	0.501887	133	28	104
**1129**	0.5	186	135	51	**2581**	0.486141	228	232	9
**1179**	0.488701	173	117	64	**2585**	0.524	131	13	106
**1185**	0.571	348	372	−111	**2612**	0.504792	158	76	79
**1187**	0.510989	186	127	51	**2618**	0.489028	156	82	81
**1249**	0.497222	179	123	58	**2717**	0.555556	190	105	47
**1274**	0.502	251	263	−14	**2732**	0.571	140	8	97
**1315**	0.514368	179	111	58	**2751**	0.490667	184	138	53
**1391**	0.494005	206	180	31	**2786**	0.562	140	12	97
**1401**	0.490446	154	77	83	**2831**	0.603	155	20	82
**1411**	0.491803	180	129	57	**2853**	0.52214	283	305	−46
**1444**	0.495238	156	78	81	**2861**	0.522822	252	245	−15
**1458**	0.5	166	95	71	**2876**	0.635	223	114	14
**1478**	0.558074	197	116	40	**2877**	0.519651	238	221	−1
**1587**	0.485849	206	187	31	**2879**	0.7	168	3	69
**1595**	0.547414	127	−5	110	**2884**	0.486425	215	205	22
**1604**	0.489189	181	133	56	**2894**	0.494279	216	200	21
**1642**	0.603	225	136	12	**2907**	0.488889	220	213	17
**1651**	0.586	309	290	−72	**2918**	0.490028	172	114	65
**1662**	0.507576	134	27	103	**2959**	0.489362	230	233	7

**SA**: The number bits in both **SAM** and the test set. **SB**: The number bits in the test set, but not **SAM**. **SC**: The number bits in **SAM** but not the test set.

**Table 2:** Molecular descriptors of the examined 26 compounds and **SAM**.

Comp.	ALog p	MW	HBA	HBD	Rotatable Bonds	Rings	Aromatic Rings	MFPSA	Minimum Distance
**SAM**	−4.25	399.45	9	4	7	3	2	0.483	0
**50**	−1.38	297.27	9	4	3	3	2	0.508	0.768
**56**	−1.38	365.21	11	5	4	3	2	0.602	0.738
**152**	−0.77	287.21	8	3	5	2	2	0.502	0.836
**186**	−1.31	285.23	8	4	2	3	2	0.52	0.884
**190**	−1.04	435.43	11	4	7	2	1	0.576	0.874
**214**	−0.17	395.41	9	4	5	3	1	0.577	0.91
**241**	−1.88	267.24	8	4	2	3	2	0.539	0.877
**310**	−1.88	267.24	8	4	2	3	2	0.539	0.877
**1129**	−2.81	476.49	11	1	7	4	2	0.624	0.896
**1187**	−2.39	362.38	5	4	6	3	1	0.414	0.801
**1444**	−0.64	383.4	9	3	5	3	1	0.56	0.874
**1478**	−3.74	434.45	9	3	7	3	2	0.316	0.478
**1913**	−3.05	511.5	12	5	9	3	2	0.545	0.67
**1995**	−0.99	482.51	7	2	6	3	2	0.442	0.796
**2017**	−2.16	365.24	12	6	4	3	2	0.655	0.856
**2036**	−1.59	440.48	11	2	7	4	2	0.594	0.781
**2042**	−2.09	285.26	9	5	2	3	2	0.589	0.874
**2176**	−1.93	390.35	10	5	4	4	3	0.491	0.838
**2376**	−1.32	269.26	8	4	2	3	2	0.54	0.917
**2396**	−4.6	497.5	9	4	7	4	1	0.484	0.705
**2467**	−2.12	405.39	9	2	5	3	1	0.628	0.87
**2532**	−0.73	469.53	7	4	6	4	2	0.266	0.909
**2612**	−1.98	460.77	10	4	8	2	2	0.572	0.594
**2732**	−0.82	299.22	8	3	5	3	2	0.504	0.752
**2831**	−0.98	305.23	9	4	5	2	2	0.55	0.76
**2879**	−1.26	294.31	8	3	3	3	2	0.395	0.846

ALog p: lipid–water partition coefficient, MWt: molecular weight, HBA: hydrogen bond acceptor, HBD: hydrogen bond donor, Rotatable bonds: any single non-ring bond, attached to a non-terminal, non-hydrogen atom, Rings: non-aromatic rings, MFPSA: molecular fractional polar surface area, Minimum Distance: the shortest distance between a tested compound and the reference one.

**Table 3:** Binding free energies (calculated ΔG in kcal/mol) of the examined compounds and ligand SAM against 2′OMTase.

Comp.	Name	ΔG [kcal/mol]
**SAM**	*S*-Adenosylmethionine	−21.52
**50**	Arranon (Nelarabine)	−13.84
**56**	Fludara (Fludarabine)	−15.53
**152**	Tenofovir (PMPA)	−13.58
**186**	Fludarabine	−14.19
**190**	Azactam (aztreonam)	−14.88
**214**	Cefdinir (cefdinir)	−15.41
**241**	Adenosine	−14.09
**310**	VIRA-A (vidarabine)	−14.10
**1129**	Cefazolin	−16.66
**1187**	Protirelin	−18.68
**1444**	Ceftizoxime	−10.99
**1478**	Xanthinol Nicotinate	−16.19
**1913**	Calcium folinate	−19.09
**1995**	Raltegravir	−21.07
**2017**	Adenosine 5′-monophosphate	−15.34
**2036**	Ceftezole	−15.21
**2042**	Vidarabine	−13.41
**2176**	Regadenoson	−18.54
**2376**	2′-Deoxyadenosine	−13.16
**2396**	Ertapenem	−20.73
**2467**	Ceftizoxime	−13.63
**2532**	Methylergometrine	−20.46
**2612**	Thiamine pyrophosphate hydrochloride	−18.03
**2732**	Besifovir	−13.47
**2831**	Tenofovir	−14.36
**2879**	Puromycin aminonucleoside	−15.33

## Data Availability

All data is contained in the published article.

## Supplementary Materials

The following supporting information can be downloaded at: https://www.mdpi.com/article/10.3390/molecules27072287/s1, The method of Fingerprints; Molecular Similarity; Docking; Molecular dynamics; and MMPBSA studies.

## Sample Availability

Samples of the compoundsre not available from the authors.
