# Supplementary material for: Ligand and Structure-Based In Silico Determination of the Most Promising SARS-CoV-2 nsp16-nsp10 2′-o-Methyltransferase Complex Inhibitors among 3009 FDA Approved Drugs
Source: Molecules. 2022 Mar 31;27(7):2287. doi: 10.3390/molecules27072287 (PMC9000629; doi:10.3390/molecules27072287)
Supplement: Supplementary file 1 [file molecules-27-02287-s001.zip › molecules-1630696-supplementary.pdf]

Supporting Data

Ligand and structure-based *in silico* determination of the most promising SARS-CoV-2 nsp16-nsp10 2'- *O* -methyltransferase complex inhibitors among 3009 FDA approved drugs

Ibrahim. H. Eissa[a]*, Mohamed S. Alesawy [a], Abdulrahman M. Saleh [a], Eslam B. Elkaeed[b], Bshra A. Alsfouk [c], Abdul-Aziz M.M. El-Attar [d], Ahmed M. Metwaly [e,f]*,

[a] Pharmaceutical Medicinal Chemistry & Drug Design Department, Faculty of Pharmacy (Boys), Al-Azhar University, Cairo11884, Egypt.

[b] Department of Pharmaceutical Sciences, College of Pharmacy, Almaarefa University, Ad Diriyah 13713, Riyadh, Saudi Arabia.

[c] Department of Pharmaceutical Sciences, College of Pharmacy, Princess Nourah bint Abdulrahman University, P.O. Box 84428, Riyadh 11671, Saudi Arabia

[d] Pharmaceutical Analytical Chemistry Department, Faculty of Pharmacy, Al-Azhar University, 11884, Nasr City, Cairo, Egypt

[e] Pharmacognosy and Medicinal Plants Department, Faculty of Pharmacy (Boys), Al-Azhar University, Cairo 11884, Egypt

[f] Biopharmaceutical Products Research Department, Genetic Engineering and Biotechnology Research Institute, City of Scientific Research and Technological Applications (SRTA-City), Alexandria 21934, Egypt

Content

Method	Fingerprint studies
	Molecular Similarity
	Docking studies
	Molecular dynamics
	MMPBSA

Method

Fingerprint study

Fingerprint study of the selected compounds against the co-crystallized ligand of the target enzyme was carried out calculated using Discovery studio 4.0. At first, the CHARMM force field was applied then the compounds were prepared using prepare ligand protocol. Then, the compounds were used as a test set while the co-crystallized ligand was used as a reference compounds. The protocol was adjusted to give the most related compounds to the co-crystallized ligand. The default molecular properties were applied. The used fingerprints were based on some parameters related to type of atoms which may be one of the following: charge, hybridization, H-bond acceptor, H-bond donor, Positive ionizable, Negative ionizable, Halogen, Aromatic, or None of the above. In addition, it includes the ALogP category of atoms.

Molecular Similarity

Molecular Similarity of the examined compounds was carried out calculated using Discovery studio 4.0. At first, the CHARMM force field was applied then the compounds were prepared using prepare ligand protocol. Then, compounds were used as a test set while the co-crystallized ligand was used as a reference compounds. The protocol was adjusted to give 5% output. The default molecular properties were applied. The molecular properties include number of rotatable bonds, number of rings, number of aromatic rings, number of hydrogen bond donors (HBA), number of hydrogen bond acceptors (HBD), partition coefficient (ALog p), molecular weight (M. Wt), and molecular fractional polar surface area (MFPSA).

Docking studies

Crystal structure of target enzyme) was obtained from Protein Data Bank. The docking investigation was accomplished using MOE2014 software. At first, the crystal structure of the protein was prepared by removing water molecules. Only one chain was retained beside the co-crystallized ligand. Then, the selected chain was protonated and subjected to minimization of energy process. Next, the active site of the target protein was defined.

Structures of the tested compounds and the co-crystallized ligand were drawn using ChemBioDraw Ultra 14.0 and saved as MDL-SD format. Such file was opened using MOE to display the 3D structures which were protonated and subjected to energy minimization. Formerly, validation of the docking process was performed by docking the co-crystallized ligand against the isolated pocket of active site. The produced RMSD value indicated the validity of process. Finally,

docking of the tested compounds was done through the dock option inserted in compute window. For each docked molecule, 30 docked poses were produced using ASE for scoring function and force field for refinement. The results of the docking process were then visualized using Discovery Studio 4.0 software.

Molecular Dynamics (MD) Simulations

The system was prepared using the web-based CHARMM-GUI[1-3] interface with the CHARMM36 force field[4]. All the simulations were done using the NAMD 2.13[5] package. The TIP3P explicit solvation model was used[6], and the periodic boundary conditions were set with a dimension of the dimensions ---- Å, ----- Å, and ----- Å in x, y, and z, respectively. The parameters for the top docking results were generated using the CHARMM general force field[7] Afterward, the system was neutralized using ---- (Cl^-/Na^+) ions. The MD protocols involved minimization, equilibration, and production. a 2 *fs* time step of integration was chosen for all MD simulations, the equilibration was carried in the canonical (*NVT*) ensemble, while the isothermal–isobaric (*NPT*) ensemble was for the production. Through the 100 *ns* of MD production, the pressure was set at 1 atm using the Nose´–Hoover Langevin piston barostat[8, 9] with a Langevin piston decay of 0.05 *ps* and a period of 0.1 *ps*. The temperature was set at 298.15 K using the Langevin thermostat[10]. A distance cutoff of 12.0 Å was applied to short-range nonbonded interactions with a pair list distance of 16 Å, and Lennard Jones interactions were smoothly truncated at 8.0 Å. Long-range electrostatic interactions were treated using the particle-mesh Ewald (PME) method[11, 12], where a grid spacing of 1.0 Å was used for all simulation cells. All covalent bonds involving hydrogen atoms were constrained using the SHAKE algorithm[13]. For consistency, we have applied the same protocol for all MD simulations.

Binding Energy Calculations

The one-average molecular mechanics generalized Born surface area (MM/GBSA)[14, 15] approach implemented in the MOLAICAL code[16] was used for the relative binding energy calculations, in which the ligand (L) binds to the protein receptor (R) to form the complex (RL),

$$\Delta G_{bind} = \Delta G_{RL} - \Delta G_{R} - \Delta G_{L}$$

which can be represented by contributions of different interactions,

$$\Delta G_{bind} = \Delta H - T\Delta S = \Delta E_{MM} + \Delta G_{Sol} - T\Delta S$$

where the changes in the gas phase molecular mechanics (ΔE_{MM}), solvation Gibbs energy (ΔG_{Sol}), and conformational entropy $(-T\Delta S)$ are determined as follows: ΔE_{MM} is the sum of the changes in the electrostatic energies ΔE_{ele}, the van der Waals energies ΔE_{vdW}, and the internal energies ΔE_{int} (bonded interactions); ΔG_{Sol} is the total of both the polar solvation (calculated using the generalized Born model) and the nonpolar solvation (calculated using the solvent-accessible surface area) and $-T\Delta S$ is calculated by the normal mode analysis. The solvent dielectric constant of 78.5 and the surface tension constant of 0.03012 kJ mol^{-1} $Å^2$ were used for MM/GBSA calculations.
